# Large-Area MoS_2_ Films Grown on Sapphire and GaN Substrates by Pulsed Laser Deposition

**DOI:** 10.3390/nano13212837

**Published:** 2023-10-26

**Authors:** Marianna Španková, Štefan Chromik, Edmund Dobročka, Lenka Pribusová Slušná, Marcel Talacko, Maroš Gregor, Béla Pécz, Antal Koos, Giuseppe Greco, Salvatore Ethan Panasci, Patrick Fiorenza, Fabrizio Roccaforte, Yvon Cordier, Eric Frayssinet, Filippo Giannazzo

**Affiliations:** 1 Institute of Electrical Engineering, Slovak Academy of Sciences, Dúbravská cesta 9, 84104 Bratislava, Slovakia; stefan.chromik@savba.sk (Š.C.); edmund.dobrocka@savba.sk (E.D.); lenka.pribusova-slusna@savba.sk (L.P.S.); marcel.talacko@savba.sk (M.T.); 2 Faculty of Mathematics, Physics and Informatics, Comenius University Bratislava, 84248 Bratislava, Slovakia; maros.gregor@fmph.uniba.sk; 3 HUN-REN Centre for Energy Research, Institute of Technical Physics and Materials Science, Konkoly-Thege ut 29-33, 1121 Budapest, Hungary; pecz.bela@ek.hun-ren.hu (B.P.); koos.antal@ek.hun-ren.hu (A.K.); 4 Consiglio Nazionale delle Ricerche—Istituto per la Microelettronica e Microsistemi (CNR-IMM), Strada VIII 5, 95121 Catania, Italy; giuseppe.greco@imm.cnr.it (G.G.); salvatoreethan.panasci@imm.cnr.it (S.E.P.); patrick.fiorenza@imm.cnr.it (P.F.); fabrizio.roccaforte@imm.cnr.it (F.R.); filippo.giannazzo@imm.cnr.it (F.G.); 5 CNRS, CRHEA, Université Côte d’Azur, 06560 Valbonne, France; yvon.cordier@crhea.cnrs.fr (Y.C.); eric.frayssinet@crhea.cnrs.fr (E.F.)

**Keywords:** MoS_2_, GaN, sapphire substrates, pulsed laser deposition, structural properties, electrical properties

## Abstract

In this paper, we present the preparation of few-layer MoS_2_ films on single-crystal sapphire, as well as on heteroepitaxial GaN templates on sapphire substrates, using the pulsed laser deposition (PLD) technique. Detailed structural and chemical characterization of the films were performed using Raman spectroscopy, X-ray photoelectron spectroscopy, X-ray diffraction measurements, and high-resolution transmission electron microscopy. According to X-ray diffraction studies, the films exhibit epitaxial growth, indicating a good in-plane alignment. Furthermore, the films demonstrate uniform thickness on large areas, as confirmed by Raman spectroscopy. The lateral electrical current transport of the MoS_2_ grown on sapphire was investigated by temperature (T)-dependent sheet resistance and Hall effect measurements, showing a high n-type doping of the semiconducting films (n_s_ from ~1 × 10^13^ to ~3.4 × 10^13^ cm^−2^ from T = 300 K to 500 K), with a donor ionization energy of E_i_ = 93 ± 8 meV and a mobility decreasing with T. Finally, the vertical current injection across the MoS_2_/GaN heterojunction was investigated by means of conductive atomic force microscopy, showing the rectifying behavior of the I-V characteristics with a Schottky barrier height of ϕ_B_ ≈ 0.36 eV. The obtained results pave the way for the scalable application of PLD-grown MoS_2_ on GaN in electronics/optoelectronics.

## 1. Introduction

Two-dimensional layered (2D) transition metal dichalcogenides (TMDs), especially molybdenum disulfide (MoS_2_), have attracted widespread attention due to their unique electronic, mechanical, and optical properties. Layered MoS_2_ consists of a vertical stack of single layers 0.65 nm thick which are held together by van der Waals interactions. Depending on the number of MoS_2_ monolayers, it can be possible to tune the bandgap of MoS_2_. Monolayer MoS_2_ exhibits a large direct bandgap of about 1.8 eV [1], resulting in enhancement in the carrier mobility, which can be utilized for potential applications in next-generation electronic and optoelectronic [2,3,4,5] devices. Conventional approaches, such as Scotch-tape-assisted micromechanical exfoliation [6,7] or chemical exfoliation [8], are not suitable for large-area device applications. The chemical vapor deposition (CVD) technique is considered one of the promising techniques for the growth of thin MoS_2_ films [9,10]. However, it has some limitations, such as the precise control of the thickness uniformity, the substrate temperature, and the use of multiple precursors [11]. One of the typical physical vapor deposition methods for the fabrication of 2D layered materials is pulsed laser deposition (PLD), which offers several advantages—the capability to produce large-area, few-monolayer-thick, highly crystalline MoS_2_ films at relatively low substrate temperatures (the typical substrate temperature for PLD growth is 700 °C), as well as the stoichiometric transfer of the ablated material from the target to the substrate [12,13,14]. In these studies, 2D MoS_2_ thin films have been grown on Al_2_O_3_, GaN, and SiC-6H substrates. C-plane sapphire (Al_2_O_3_ (0001)) wafers are the most commonly employed substrates for growth of MoS_2_-based 2D materials. This material is a chemically stable hexagonal single crystal, with 60°-rotation symmetry about its c-axis. GaN is a wide bandgap semiconductor and the ideal material of choice for high-frequency transistors (GaN HEMTs technology) and power-switching devices [15,16,17], as well as for optoelectronic devices (LEDs and laser diodes). MoS_2_ and GaN are suitable candidates for 2D/3D heterostructures, as low lattice parameter mismatch between these two materials (<1%) and similar thermal expansion coefficients promise high-quality epitaxial alignment between the materials [18,19,20,21].

In this article, we demonstrate the large-area PLD growth of highly uniform, few-monolayer-thick MoS_2_ films on c-plane sapphire and heteroepitaxial GaN/sapphire substrates. The films are of excellent crystalline quality and thickness homogeneity, and we were able to prepare them on large-area surfaces, increasing the possibility of using these films for a wide range of applications. Noteworthily, and differently than in previous works [12] where S-enriched MoS_2_ targets were used to deposit MoS_2_ films with the highest degree of crystallinity but with excess S content, we succeeded in depositing stoichiometric MoS_2_ films from a target with a Mo:S ratio of 1:2. Furthermore, thanks to the absence of MoO_3_ components in the as-deposited MoS_2_ films, no need for additional post-deposition annealing in S is necessary to restore MoS_2_ stoichiometry, in contrast to a finding reported in an earlier study [14]. We employed various characterization techniques to investigate the structural and electrical properties of the prepared films. In particular, we provided a detailed analysis of the orientation relationship between the MoS_2_ film and the c-sapphire and GaN substrates, for which few results are available in the literature. Furthermore, we provided a detailed electrical investigation of the in-plane transport properties of PLD-grown MoS_2_ films on insulating sapphire substrates, extracting key physical parameters like the ionization energy of donors and providing insight into the limiting mechanisms of carrier mobility. To the best of our knowledge, this type of investigation has been not reported so far in PLD-grown MoS_2_. The results are useful for benchmarking the electronic quality of the material produced by this approach compared to that of MoS_2_ produced by alternative methods.

## 2. Materials and Methods

Two types of wafers, i.e., single-polished 10 × 10 mm^2^ and 0.5 mm thick c-oriented ((0001) plane) sapphire and GaN (0001)/c-sapphire substrates, were used for the MoS_2_ growth. The epitaxial GaN films with thicknesses of 4 μm were grown by means of metal–organic chemical vapor deposition on c-sapphire substrates [22].

Few-monolayer (ML)-thick MoS_2_ films were prepared via PLD (MBE/PLD-2000 system). The setup consists of a KrF excimer laser with a 248 nm wavelength, mirrors, and a focusing lens. An energy laser pulse of 70 mJ, with a repetition rate of 4 Hz and a laser spot size of 2 mm^2^, was applied during the growth process. As a target, a commercially purchased stoichiometric MoS_2_ two inches in diameter was used. The target was rotated at a speed of 6 rpm and rastered to avoid pit formation. The distance from the target to the substrate was optimized at 10 cm. The substrate was heated by irradiation from the SiC heater, eliminating the need for direct contact between the heater and the substrate. This is a definite advantage as it allows us to rotate the substrates, enhancing the homogeneity of the deposited films, and the samples do not require any clamping or silver paste. The film growth was carried out at a deposition temperature of 860 °C under 2 × 10^−6^ Torr background pressure. The 860 °C was the temperature of the thermocouple, which was placed close to the SiC heater so it would show the actual temperature T_S_ for a non-transparent Si substrate. In the case of a transparent sapphire substrate, this temperature was about 160 °C lower, i.e., in our case, T_S_~700 °C. After the film growth, the samples were cooled down to 200 °C at a rate of 50 °C/min before allowing for natural cooling. The final thickness of the MoS_2_ films was controlled by the number of pulses; typically, it was in the range between 400 and 800 pulses.

Raman spectroscopy was performed using an Alpha 300R micro-Raman system with a 532 nm excitation laser. The laser beam was focused by a 50× objective lens with a numerical aperture of 0.8. The acquisition time was 5 s, and the laser power was kept below 2 mW for all measurements. All of the spectra were acquired in ambient conditions.

X-ray photoelectron spectroscopy (XPS—Omicron multiprobe system with hemispherical analyzer, Scienta Omicron, Taunusstein, Germany) was performed using monochromatic Al K-alpha X-rays (1486.6 eV). All spectra were measured at an ambient temperature, with photoemission of 45° from the surface.

A Bruker D8 DISCOVER diffractometer equipped with a rotating Cu anode operating at 12 kW was used to determine the crystallographic orientation perpendicular to the film’s surface (symmetric 2θ/ω configuration). To specify the in-plane orientation of the MoS_2_ with respect to the substrate, ϕ-scans were carried out. All X-ray measurements were performed in parallel-beam geometry with a Goebel mirror in the primary beam.

High-resolution transmission electron microscopy (HR TEM) analyses were carried out with an aberration-corrected Thermofisher Themis 200 microscope (Thermo Fisher Scientific Inc., Waltham, MA, USA). Cross-sectional TEM specimens of MoS_2_/sapphire and MoS_2_/GaN/sapphire were prepared by a Ga FIB (focused ion beam) and finished at 2 kV. During the FIB process, a platinum coating was deposited in order to defend the lamella from the energetic gallium ions. We also evaporated an amorphous carbon layer, as we wanted to avoid close contact between the MoS_2_ and platinum particles.

The sheet resistance, carrier density, and mobility of the MoS_2_ grown on the sapphire substrate were evaluated by four-point probe and Hall effect measurements in the van der Pauw configuration. For this purpose, Ni(20 nm)/Au(80 nm) contacts were deposited by sputtering at the four corners of square-shaped (1 cm × 1 cm) MoS_2_/sapphire samples. Ohmic contacts on MoS_2_ were obtained with as-deposited Ni/Au stacks. The vertical current injection at the MoS_2_/GaN interface was probed at the nanoscale by conductive atomic force microscopy (C-AFM) with a DI3100 system by Bruker with Nanoscope V electronics, using Pt-coated conductive tips.

## 3. Results

**Figure 1:**
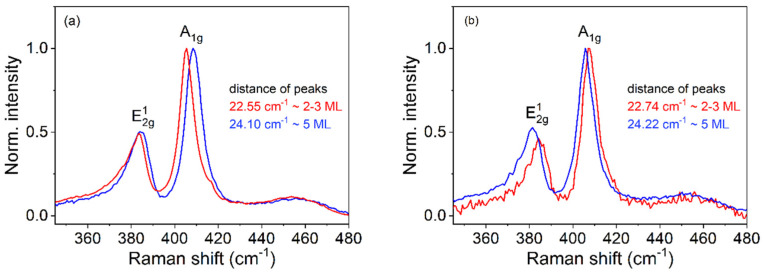
Normalized Raman spectra of MoS_2_ deposited on sapphire (**a**) and on GaN/sapphire (**b**), with various pulse numbers. The two characteristic in-plane (E^1^_2g_) and out-of-plane (A_1g_) vibrational modes are labeled.

We performed characterization using Raman spectroscopy in order to verify the thicknesses and properties of our MoS_2_ films (Figure 1a,b). Raman spectroscopy serves as a strong tool to determine the vibrational modes of TMDs. The characteristic Raman modes E^1^_2g_ and A_1g_ are the most intense signals for the 2H-MoS_2_ phase and can be used to determine the number of MoS_2_ layers. This number depends on the difference Δω between the E^1^_2g_ and A_1g_ peak wavenumbers. We obtained values of Δω~22.5–22.7 cm^−1^ and ~24.2 cm^−1^ for the MoS_2_ films shown in Figure 1a,b, corresponding to two to three monolayers (prepared by 400 laser pulses) and five monolayers (prepared by 800 laser pulses) of MoS_2_, respectively. The thickness uniformity of the MoS_2_ films was determined from arrays of 40 × 40 Raman spectra collected from an area measuring 10 × 10 µm.

**Figure 2:**
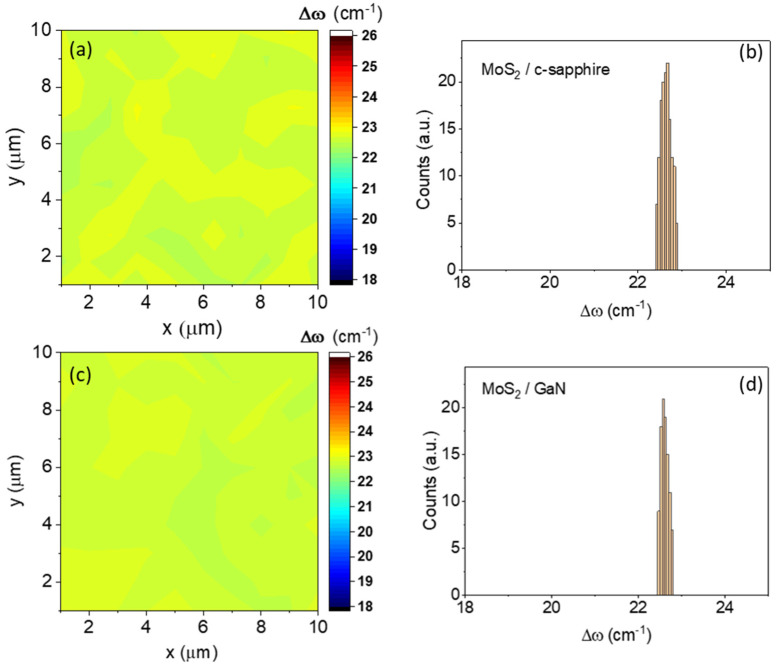
Raman mapping images and histograms of the differences (Δω) between the E^1^_2g_ and A_1g_ peaks collected on 10 µm × 10 µm scan areas on the MoS_2_ 2–3 ML in thickness and grown on c-sapphire (**a**,**b**) and (**c**,**d**) GaN/c-sapphire substrates.

Figure 2a,c show two representative color maps of the Δω values extracted from the arrays of spectra measured on MoS_2_/sapphire and MoS_2_/GaN surfaces, respectively. Figure 2b,d are the histograms of the Δω values in the two maps, from which a very narrow distribution can be observed. The same results were obtained at different positions of the samples’ surfaces, demonstrating a high degree of thickness uniformity over a large area. Raman analyses demonstrated that MoS_2_ films with nearly identical thicknesses were obtained by PLD on the sapphire and GaN/sapphire substrates.

**Figure 3:**
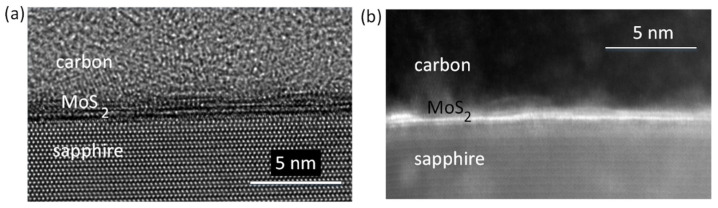
(**a**) HREM image in TEM mode shows 2–3 ML of the MoS_2_ grown on c-sapphire. (**b**) In the HAADF STEM image mode, the Mo appears very bright (**b**).

**Figure 4:**
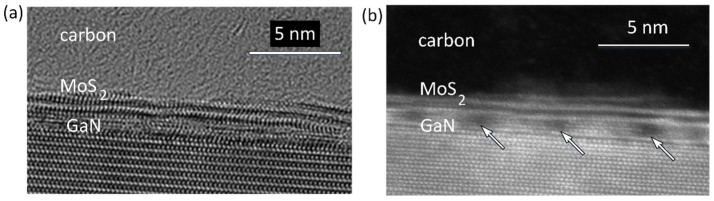
HREM TEM (**a**) and HAADF STEM (**b**) images of the MoS_2_ 2–3 ML in thickness and grown on GaN/c-sapphire. Locally, there are other small GaN islands on the surface of the perfect GaN covered by the MoS_2_. The observed periodic dark spots, denoted by arrows, indicate empty voids between the islands.

High-resolution images taken in TEM mode and based on the lattice spacings show 2–3 monolayers of MoS_2_ grown on c-sapphire, as well as on GaN/c-sapphire (Figure 3a and Figure 4a). Figure 3b and Figure 4b show cross-sectional scanning transmission electron microscopy (STEM) images taken with a high-angle annular dark field (HAADF) detector, called Z contrast. In the HAADF STEM image mode, Mo atoms, possessing a higher atomic number Z, exhibit significantly enhanced brightness, allowing for the detection of MoS_2_ layers. The image in Figure 4b shows 2–3 ML of MoS_2_ in close contact to GaN; there is no oxide or amorphous layer on the top of GaN.

However, the periodic darker regions observed inside the GaN suggest the presence of potential damage or structural imperfections.

**Figure 5:**
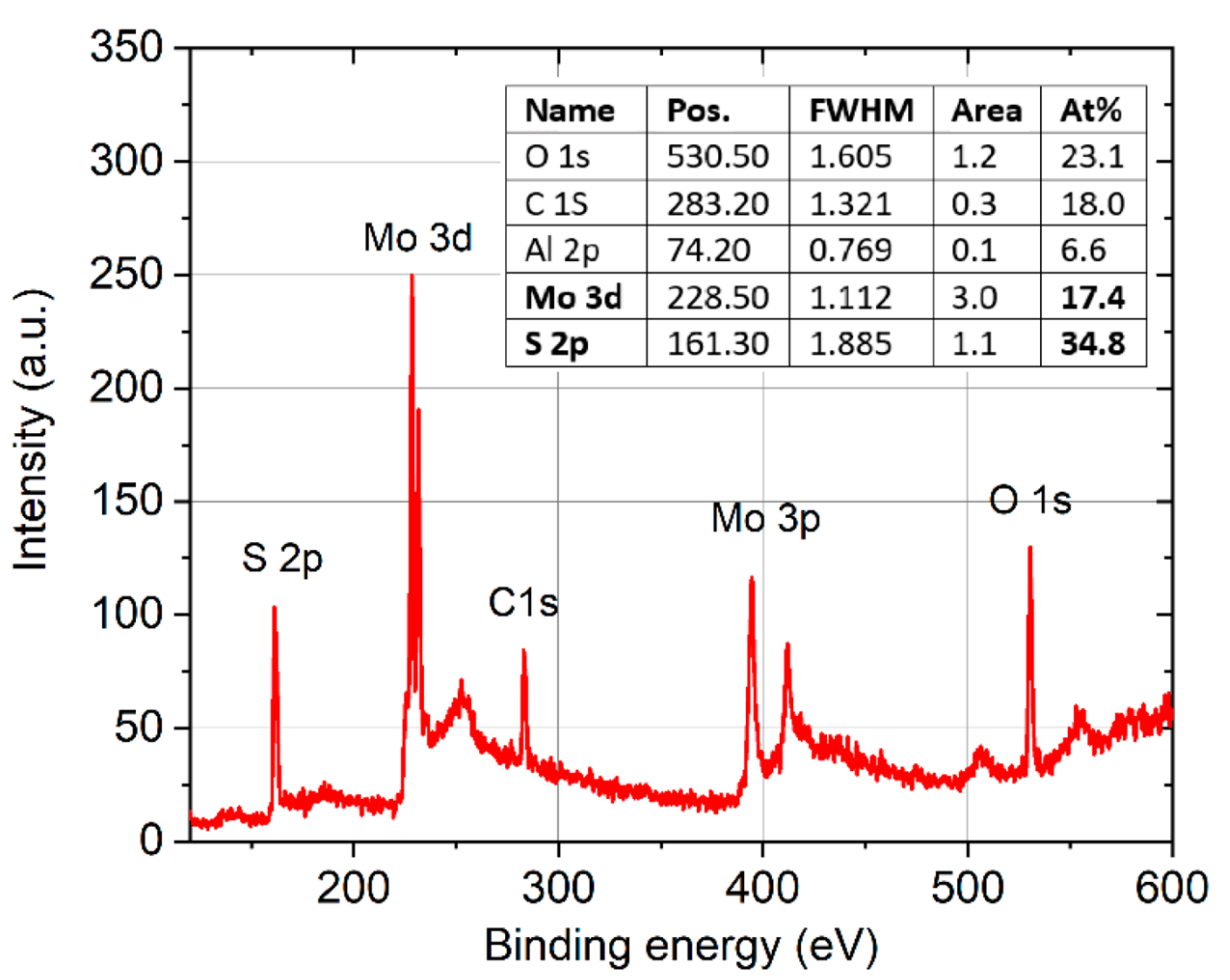
Typical XPS survey spectrum of an MoS_2_/c-sapphire. The films were deposited in a stoichiometric composition (Mo:S  =  1:2) without any significant chemical shift (inset).

**Figure 6:**
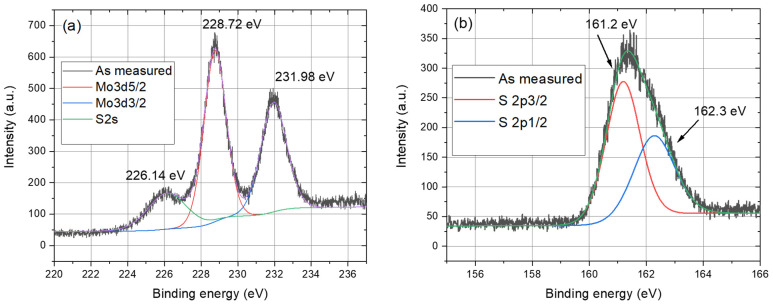
Deconvolutions of XPS Mo 3d (**a**) and S 2p (**b**) spectra of a 3-monolayer-thick MoS_2_ grown on c-sapphire.

XPS was used to measure the chemical composition and stoichiometry of the MoS_2_ samples. The evaluated atomic concentrations obtained from the survey spectrum confirmed the ideal stoichiometry of the MoS_2_ films grown on both types of substrates, and we registered carbon on the top of the surface originating from elements of the atmosphere and substrate (Figure 5). The Shirley background subtraction procedure was applied on the high-resolution spectra taken from the samples, and peak fitting was carried out through the selection of Gaussian–Lorentzian functions (Figure 6).

Peak deconvolutions of the spectra revealed binding energies at 228.72 eV and 231.98 eV, corresponding to Mo^4+^ 3d_5/2_ and 3d_3/2_ orbitals of 2H-MoS_2_, respectively. The peak at 226.14 eV was assigned to the S 2s orbital. Additional peaks were observed from the doublet peak of MoS_2_, S 2p_3/2_, and S 2p_1/2_ detected at 161.2 eV and 163.2 eV, respectively. The presence of the Mo 3d and S 2p states confirmed the formation of 2H MoS_2_ [23].

**Figure 7:**
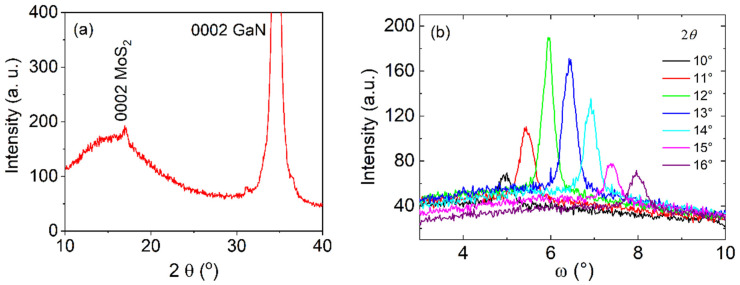
2θ/ω diffraction pattern of a 5-monolayer-thick MoS_2_ film deposited on GaN/c-sapphire (**a**). Rocking curves taken from the 0002 diffraction of the MoS_2_ at different 2θ values (**b**). The FWHM of the rocking curves reached a value of about 0.3–0.4°. (FWHM 0002 GaN = 0.11 °).

**Figure 8:**
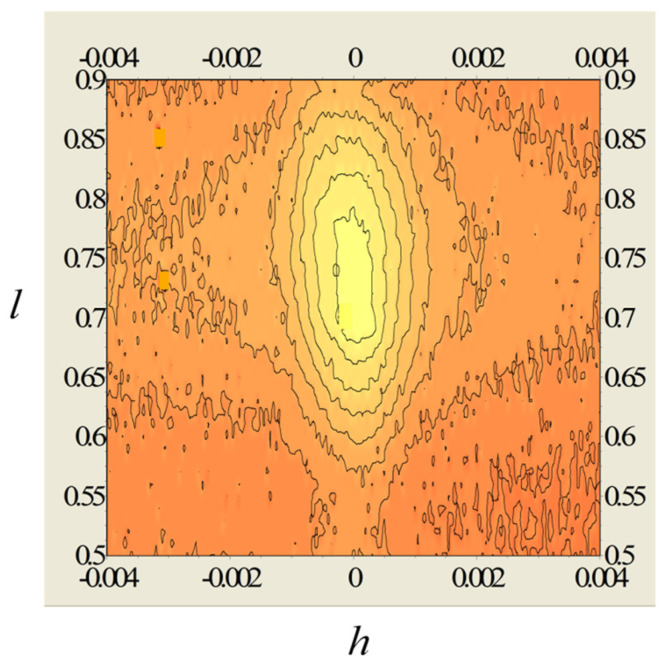
Reciprocal space maps around 0002 Bragg diffraction of the MoS_2_ on GaN/c-sapphire for ϕ = 0°.

XRD analyses confirmed the preferential growth (with the c-axis perpendicular to the substrate plane) of the MoS_2_ films grown on c-sapphire as well as on GaN/c-sapphire substrates (Figure 7a). The standard 2θ/ω diffraction pattern presented in Figure 7a reveals an extremely wide diffraction, 0002, of MoS_2_. However, the rocking curves (Figure 7b) recorded at different values of 2θ around the tabulated value 14.378° were surprisingly narrow; the values of the full width at half maximum (FWHM) varied between 0.3° and 0.4°. This indicates a very strange shape of the 0002 diffraction spot, which was elongated in reciprocal space (RS) along the direction perpendicular to the sample’s surface. This was confirmed by an RS map of the diffraction 0002, as shown in Figure 8. The map is presented with the dimensionless linear coordinates *h* and *l*, introduced in RS in parallel and perpendicular directions with respect to the sample’s surface. They have the units $1/d_{11\bar{2}0}$ and $1/d_{0001}$, respectively, where $d_{hkil}$ is the interplanar distance of the corresponding lattice planes *hkil* of the GaN. The advantage of the parameters *h* and *l* is that they acquire integer values at the diffraction spots of the substrate and make the interpretation and evaluation of the measurements quite easy. Note that, for greater clarity and better insight, the horizontal dimension of the map was 15 times enlarged with respect to its actual value.

**Figure 9:**
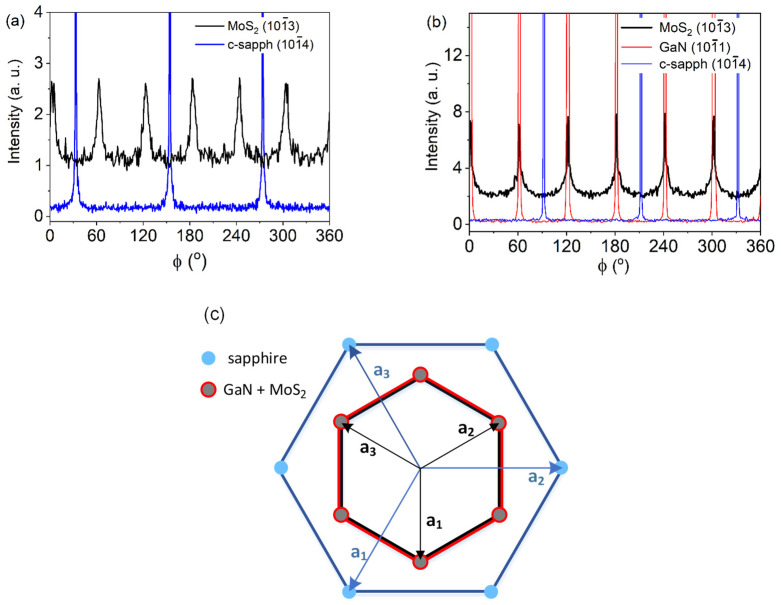
ϕ -scans of the $\left(10\bar{1}3\right)$ MoS_2_ planes. In-plane ordering observed in cases of MoS_2_ films grown on both c-sapphire (**a**) and GaN/c-sapphire (**b**) substrates. Schematic representation of the orientation relationship between c-plane sapphire, GaN, and MoS_2_. Vectors **a_1_**, **a_2_**, and **a_3_** represent crystallographic axes in the (0001) plane of the sapphire, the GaN and MoS_2_ lattice (**c**).

The in-plane orientation relationship between the film and the substrate was examined through ϕ-scans of the strongest diffraction, $10\bar{1}3$, of the hexagonal MoS_2_ (Figure 9a,b). A ϕ-scan of the $10\bar{1}4$ diffraction of the sapphire was also recorded. Six maxima of MoS_2_ were detected, indicating the presence of a biaxial texture. The hexagonal MoS_2_ lattice was rotated by an angle of 30° to the sapphire lattice (Figure 9a), and the crystallographic orientation can be described as MoS_2_ (0001) $\left[10\bar{1}0\right]$ ‖ sapphire (0001) $\left[2\bar{1}\bar{1}0\right]$. A similar situation occurred in the case of MoS_2_ grown on the GaN/c-sapphire substrate. To reveal the orientation relationship of the in-plane ordered MoS_2_ with respect to the GaN/c-sapphire, the ϕ-scan from the $10\bar{1}1$ diffraction of the GaN layer was measured beside the ϕ-scan of the $10\bar{1}4$ diffraction of the sapphire (Figure 9b). In this case, the in-plane relationship between the MoS_2_ and the GaN/c-sapphire substrate (Figure 9c) was MoS_2_ (0001) $\left[10\bar{1}0\right]$ ‖ GaN (0001) $\left[10\bar{1}0\right]$ ‖ sapphire (0001) $\left[2\bar{1}\bar{1}0\right]$. To evaluate the “perfection” of the in-plane ordering, the FWHM values of the φ-scans of the $10\bar{1}3$ MoS_2_ diffraction were estimated for the sapphire and the GaN/sapphire substrates. The obtained FWHM values were 9° and 8°, respectively, indicating the similar quality of the MoS_2_ films grown on different substrates. For reference, the FWHMs of the φ-scans for the $10\bar{1}4$ sapphire and the $10\bar{1}1$ GaN diffractions were approximately 0.2° and 0.3°, respectively.

**Figure 10:**
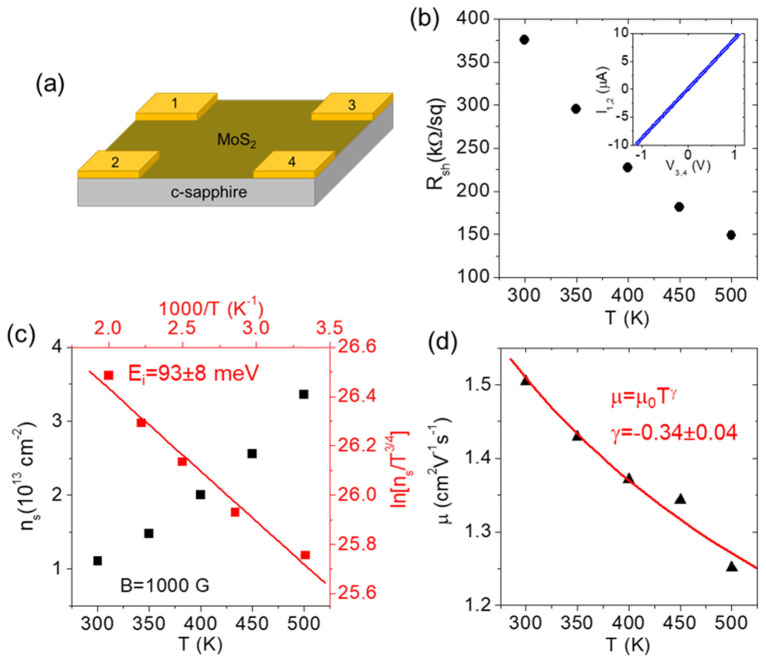
Schematic of the van der Pauw structure used for sheet resistance and Hall effect measurement on the PLD-grown MoS_2_ film on a sapphire substrate (**a**). Temperature-dependent sheet resistance R_sh_ measurement. The ohmic behavior of the as-deposited Ni/Au contacts at the four corners of the MoS_2_ film is shown in the insert (**b**). Sheet electron density n_s_ as a function of the temperature T evaluated by Hall effect measurements, with a magnetic field B = 1000 G. Red symbols show the Arrhenius plot of ln[n_s_/T^3/4^] (right scale) vs. 1000/T (upper scale). The linear fit (red line) from which the ionization energy E_i_ = 93 ± 3 meV of the donor levels responsible for n-type doping in PLD-grown MoS_2_ was evaluated (**c**). Hall electron mobility is represented by μ as a function of temperature T, and fits with the relation μ = μ_0_T^γ^, from which γ ≈ −0.34 was evaluated (**d**).

Electrical characterizations were finally carried out on the MoS_2_ films grown on the sapphire and GaN/sapphire substrates. Firstly, the sheet resistance, carrier density, and mobility of PLD-grown MoS_2_ on the insulating sapphire substrate were evaluated by four-point probe and Hall effect measurements in the van der Pauw configuration (see the schematic in Figure 10a). After preliminarily checking that the as-deposited Ni/Au pads provided Ohmic contacts onto MoS_2_ (see the insert in Figure 10b), the temperature dependence of the sheet resistance (R_sh_) was evaluated in a range from T = 300 to 500 K. As illustrated in Figure 10b, a monotonic decrease in R_sh_ from ≈375 kΩ/sq to ≈150 kΩ/sq was observed with the increase in temperature in this range, as is consistent with the expected semiconducting behavior of the MoS_2_ film. Furthermore, the Hall effect measurements, with an applied magnetic field B = 1000 G, showed an increase in the sheet electron density from n_s_ ≈ 1 × 10^13^ cm^−2^ to n_s_ ≈ 3.4 × 10^13^ cm^−2^. This was in the same temperature range (Figure 10c, left axis) that was ascribed to the ionization of shallow donors with energy levels located below the MoS_2_ conduction band. To evaluate the ionization energy E_i_ of these donor levels (i.e., their energy distance from the bottom of the conduction band E_i_ = E_c_ − E_D_), we employed the neutrality equation for the approximation of a highly n-type doped semiconductor with a low level of acceptor compensation (N_D_ >> N_A_) [24]:

(1) $$n_{s}\approx \sqrt{\frac{N_{D}N_{c}}{2}}\exp\left(-\frac{E_{i}}{2k_{B}T}\right)\propto T^{\frac{3}{4}}\exp\left(-\frac{E_{i}}{2k_{B}T}\right)$$

Here, k_B_ is the Boltzmann constant, N_D_ is the donor density, and N_C_ is the effective electron density in the MoS_2_ conduction band, which depends on the temperature as N_c_∝T^3/2^. Such an approximation of a negligible compensation is justified, in part, by the nearly ideal stoichiometry of the PLD-grown MoS_2_, without any MoO_3_ contributions, as was deduced from the XPS analyses shown in Figure 5 and Figure 6. In fact, MoO_3_ residues in the deposited films have recently been demonstrated to act with p-type doping levels, causing a significant compensation for the natural n-type doping of MoS_2_ [25]. Based on Equation (1), an Arrhenius plot of ln[n_s_T^−3/4^] vs. 1000/T is provided in right and top axis of Figure 10c, and the ionization energy E_i_ = 93 ± 8 meV was obtained from the slope of the linear fit. Finally, the temperature dependence of the electron mobility μ was evaluated from the combination of the R_sh_ and n_s_ results, according to the relation μ = 1/(qn_s_R_sh_). A slight decrease in μ from ≈1.5 cm^2^V^−1^s^−1^ to ≈1.25 cm^2^V^−1^s^−1^ was observed by increasing T from 300 to 500 K, as illustrated in Figure 10d. To obtain a deeper insight into the scattering mechanisms responsible for this decreasing trend in mobility, a fitting of the experimental data with the power function:

μ = μ_0_T^γ^ (2)

was also carried out (red line in Figure 10d), from which a value of the exponent γ = −0.34 ± 0.04 was deduced. According to the literature results, the electron mobility in MoS_2_ films is limited by two main scattering mechanisms depending on the sample temperature, i.e., the charged impurity scattering (which dominates at lower temperatures) and phonon scattering (which dominates at higher temperatures). Both the charged-impurities-limited mobility (μ_CI_) and phonon-limited mobility (μ_ph_) contributions are typically described by a power function of the temperature T (see Equation (2)), with positive and negative γ values, respectively [26]. In particular, the theoretically predicted values of γ for optical-phonon-limited mobility could range from γ = −1.69 for monolayer MoS_2_ to γ = −2.6 for bulk crystals [27]. Clearly, the negative value of γ= −0.34, obtained by fitting of the experimental results, was significantly lower than these theoretical expectations. This indicates that, although phonon scattering played an important role, other competitive mechanisms contributed to limiting the electron mobility of our PLD-grown MoS_2_. These may include not only charged impurity scattering, but also scattering by defects (such as grain boundaries of MoS_2_ domains), which can account for this lower temperature dependence of electron mobility.

**Figure 11:**
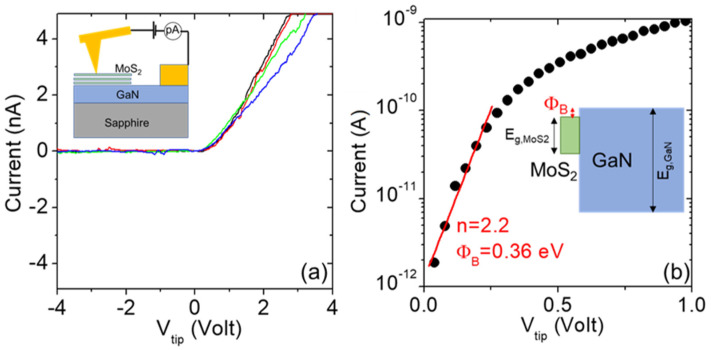
(**a**) Local I-V_tip_ characteristics collected by C-AFM at different positions on the MoS_2_/GaN heterostructures. Insert: schematic of the C-AFM setup for local current measurements. (**b**) Semi-log scale plot of a representative I-V_tip_ characteristic in the low-forward-bias regime, and fitting with the thermionic emission equation. Insert: schematic band diagram of the MoS_2_/GaN heterojunction.

After investigating the lateral current transport in the thin MoS_2_ films supported by the insulating sapphire substrate, we evaluated the vertical current injection across the 2D/3D semiconductor heterojunction formed by n-type MoS_2_ with n-type GaN. To this aim, local current-voltage (I-V_tip_) characteristics were acquired by applying a dc bias ramp between a nanometric-sized (r_tip_ = 5nm) Pt-coated AFM tip in contact with MoS_2_ and a large metal contact, consisting of a Ni(20 nm)/Au(80 nm) stack, deposited on the bare GaN surface, as is schematically illustrated in the inset of Figure 11a. This Ni/Au stack was not subjected to any thermal annealing in order to avoid the eventual structural modification of MoS_2_. In this configuration, for geometric reasons, the I-V characteristics were sensitive to local current injection through the Pt/MoS_2_/GaN vertical heterojunction, whereas current flow in the GaN layer and through the large metal contact introduced a series resistance contribution. Figure 11a shows a set of four I-V characteristics collected at different positions on the deposited MoS_2_ film. All of the curves exhibited rectifying behavior, with negligible current under negative polarization and current onset at the same forward bias. These characteristics are very reproducible under negative and small positive bias (V_tip_ < 1V), indicating the high uniformity of the MoS_2_/GaN heterostructure. The main difference can be observed at a higher positive bias, which is related to series resistance contributions. A representative I-V curve is presented on a semi-log scale at a low forward bias in Figure 11b, from which a linear increase in the current over two orders of magnitude can be observed from V = 0 to 0.25 V. This is followed by a bending, which is associated with series resistance. In this low-bias regime, we can assume that current injection from the Pt tip to MoS_2_ can be ruled by direct tunneling (assumption justified by the high doping of MoS_2_ deduced in Figure 10c), whereas the MoS_2_ heterojunction with n-GaN can be described as a Schottky junction using the thermionic emission equation:

(3) $$I=A^{*}A_{\mathrm{tip}}\exp\left(-\frac{q\phi_{B}}{k_{B}T}\right)\exp\left(\frac{{\mathrm{qV}}_{\mathrm{tip}}}{{\mathrm{nk}}_{B}T}\right)$$

In this equation, A_tip_ is the tip contact area, A* = 26.9 A cm^−2^ K^−2^ is the Richardson constant of GaN, k_B_ is the Boltzmann constant, ϕ_B_ is the Schottky barrier height at the MoS_2_/GaN interface, and n is the ideality factor. In particular, a value of n = 2.2 was extracted from the slope of the linear fit in Figure 11b, and a barrier height of ϕ_B_ = 0.36 eV was evaluated from the intercept with the current axis. This ϕ_B_ value corresponds to a small conduction band discontinuity between n-type MoS_2_ and n-GaN, as is consistent with the results reported in several recent investigations of this heterostructure [28,29]. The deviation of the ideality factor from n = 1 indicates the presence of non-idealities at the heterojunction.

## 4. Conclusions

In summary, we grew large-area epitaxial MoS_2_ films from 2 to 5 monolayers thick on c-sapphire and on GaN/c-sapphire substrates by means of pulsed laser deposition. The high thickness uniformity of the MoS_2_ was demonstrated by Raman intensity mapping. The atomic concentrations determined from the XPS survey spectrum confirmed the ideal stoichiometry of the films. The 2θ/ω diffraction revealed the growth of the MoS_2_ with the c-axis perpendicular to the substrate plane, and the FWHM values measured on the 0002 MoS_2_ diffraction were in the range of 0.3–0.4°. The ϕ-scan measurements showed a high degree of in-plane ordering in both the MoS_2_ films grown on c-sapphire as well as on GaN/c-sapphire substrates. Temperature-dependent characterization of the sheet resistance, carrier density, and mobility on MoS_2_ deposited on the sapphire substrate provided insights into the current transport mechanisms in these ultra-thin films, such as the ionization energy of the donors and the scattering phenomena limiting carrier mobility. Finally, we used C-AFM to measure the vertical current across the MoS_2_/GaN heterojunctions and observed a rectifying I-V behavior, with a low Schottky barrier of ϕ_B_ = 0.36 eV, which was estimated by fitting these curves under forward polarization of the tip. Current–voltage characteristics collected at different positions on the film indicated the high uniformity of the MoS_2_/GaN heterostructure. The obtained results represent significant progress in the advancement of large-area device fabrication and application.

## Data Availability

The data are available upon reasonable request from the corresponding author.
